# F54. PHARMACOLOGICAL ENHANCEMENT OF COGNITION AND SOCIAL COGNITION IN THE PSYCHOSIS SPECTRUM

**DOI:** 10.1093/schbul/sby017.585

**Published:** 2018-04-01

**Authors:** M Mercedes Perez-Rodriguez, Daniel Rosell, Scott Moeller, Prantik Kundu, Margaret McCLure, Erin Hazlett, Antonia New

**Affiliations:** Icahn School of Medicine at Mount Sinai

## Abstract

**Background:**

Abnormalities in cognition and social cognition represent a core feature of the schizophrenia spectrum disorders. Schizotypal personality disorder (SPD) is a milder disorder within the schizophrenia spectrum, characterized by attenuated, schizophrenia-like traits without overt psychosis.

Study 1: Working memory impairments are a core cognitive deficit in schizophrenia and SPD. The dopamine D1 receptor is a promising target to enhance working memory. We aimed to test the effect of the D1 agonist dihydrexidine (DAR-0100A) to enhance working memory in patients with SPD.

Study 2: Oxytocin modulates social cognition. However, oxytocin’s effect on social cognitive errors in the schizophrenia spectrum remains unexplored. We aimed to: 1) characterize social cognitive (mentalizing) errors in SPD patients and test their relationship with positive and negative symptoms of psychosis; 2) test the effect of intranasal oxytocin on mentalizing errors.

**Methods:**

Study 1: We performed a randomized, double blind, placebo-controlled trial of DAR-0100A (15 mg/150 ml of normal saline i.v. over 30 min) in medication-free SPD patients (n=16). Study 2: Subjects: 15 SPD patients, 15 healthy controls [HC], and 15 psychiatric controls (PC). Intervention: intranasal oxytocin 24/40IU/placebo. Measures: Movie for the Assessment of Social Cognition (MASC), a naturalistic video task measuring mentalizing accuracy, “no mentalizing” errors, “hypomentalizing” errors and “hypermentalizing” errors. The “hyper-hypomentalizing ratio” can be computed to capture the predominant mentalizing tendency; PANSS; Schizotypal Personality Questionnaire, SPQ. Mentalizing measures were compared across groups (SPD, HC, PC), and treatments (oxytocin 24IU/40IU vs placebo) using ANOVA. Pearson correlations assessed the relationship between social cognition and symptoms.

**Results:**

Study 1: Treatment with dihydrexidine (DAR-0100A) was associated with significantly improved working memory performance relative to placebo, with a very large effect size (Cohen’s d=1.14). Study 2: SPD patients had lower mentalizing accuracy (F=10.11;df=1;p=0.003), made more “No mentalizing” or “hypomentalizing” errors (F=12.92;df=1;p=0.001), and had lower hyper-hypomentalizing ratios than HCs (F=2.84; df=1;p=0.099,trend level). In a subset of patients –including 8 SPD-, a single dose of intranasal oxytocin significantly increased the hyper-hypomentalizing ratio (F=6.84, df=1,p=0.019) and increased visual attention to social cues.

“No mentalizing” and “hypomentalizing” errors were significantly correlated with negative symptoms. “Hypermentalizing” errors were significantly correlated with positive symptoms and the “ideas of reference” and “suspiciousness” SPQ subscales.

**Discussion:**

Study 1: These preliminary findings lend further clinical support to the potential of D1 receptor agonists to treat schizophrenia-spectrum working memory impairments.

Study 2: As hypothesized, SPD patients had impaired, less accurate social cognition, and made more “no mentalizing” and “hypomentalizing” errors, correlated with negative symptoms. Conversely, “hypermentalizing errors” were correlated with positive symptoms. Oxytocin increased the tendency to hypermentalize. This effect may normalize the abnormalities found at baseline in SPD patients. These results support the role of social cognitive impairments as an underlying factor of positive and negative symptoms of psychosis, with specific associations with paranoid and delusional traits. Our results also suggest that intranasal oxytocin modulates social cognitive errors in the psychosis spectrum.

